# Three-dimensional sonographic findings of diprosopus: a case report and literature review

**DOI:** 10.1186/s12884-025-07168-0

**Published:** 2025-01-20

**Authors:** Qiao Zhou, Enbo Sha, Qian Ding, Chunli Jing

**Affiliations:** Department of Obstetric and Gynecological Ultrasound, The Second Affiliated Hospital of Dalian Medical University, Dalian, China

**Keywords:** Diprosopus, Prenatal ultrasound examination, Three-dimensional ultrasound imaging, Case report

## Abstract

**Background:**

Diprosopus is one of the rarest anomalies. It typically manifests as bilateral alterations and often involves anomalies within the cranial structures. In this report, we present a case of a fetus with diprosopus diagnosed prenatally. Along with reviewing relevant literature on prenatal ultrasound diagnosis of diprosopus, we aim to raise awareness of its ultrasound characteristics.

**Case presentation:**

We report a case of craniofacial and intracranial abnormalities detected during a 26-week ultrasound examination. Two-dimensional ultrasound (2D ultrasound) demonstrates significant increases in head circumference, widening of the interocular distance, and abnormal echo patterns in the facial structure. Three-dimensional ultrasound (3D ultrasound) revealed the presence of three eye sockets (the lateral eye sockets contained eyeballs, while the central region exhibited fusion without visible eyeballs), two noses, and two mouths, with no abnormalities observed in other areas. The ultrasound findings suggested diprosopus. Following risk counseling at the prenatal diagnosis center, the pregnant woman decided to induce labor. The newborn passed away thirty minutes after delivery. The facial features of the newborn were consistent with the 3D ultrasound imaging, and the appearance of the trunk and limbs was normal. Both CT and MRI scans confirmed the diagnosis of diprosopus.

**Conclusion:**

The prenatal 2D ultrasound revealed intracranial and facial abnormalities in the fetus. 3D ultrasound imaging clearly displayed the facial duplication anomalies, highlighting the advantages of 3D ultrasound in diagnosing diprosopus. We hope to raise awareness of this rare condition and provide insights into prenatal ultrasound diagnosis through this case.

**Supplementary Information:**

The online version contains supplementary material available at 10.1186/s12884-025-07168-0.

## Background

Diprosopus is one of the rarest anomalies in humans, particularly in monoamniotic-monochorionic (MCMA) twin pregnancies [1]. The etiology remains unclear, but studies have shown a high risk of associated developmental abnormalities in the central nervous system, cardiovascular system, respiratory system, gastrointestinal system, genitourinary system, and musculoskeletal system [1–3]. In this case, the abnormalities were limited to the craniofacial and intracranial regions.

## Methods

### Data sources

Clinical data for this case were obtained from the electronic medical database of the Second Affiliated Hospital of Dalian Medical University. GE Voluson E8 ultrasound diagnostic instrument with a 3D volume probe (frequency range: 2.5 ~ 5.0 MHz) was used in this study.

### Search strategy

A search was conducted on PubMed using keywords: (“diprosopus” OR “craniofacial duplication”) AND (“Prenatal Ultrasound” OR “Obstetric Ultrasound” OR “Fetal Ultrasound” OR “Prenatal Diagnosis” OR “Fetal Ultrasonography”).

## Case presentation

The pregnant woman was a 29-year-old woman, gravida 1, parity 0, with no history of exposure to harmful substances, no past medical history, and no family history. She did not take any medications during pregnancy and reported a stable emotional state. Routine ultrasounds and prenatal checks at 6, 12, and 17 weeks showed no abnormalities. At 24 weeks of gestation, ultrasound examination revealed head and facial anomalies, leading to a referral to our hospital at 26 weeks and 4 days of gestation.

**Fig. 1:**
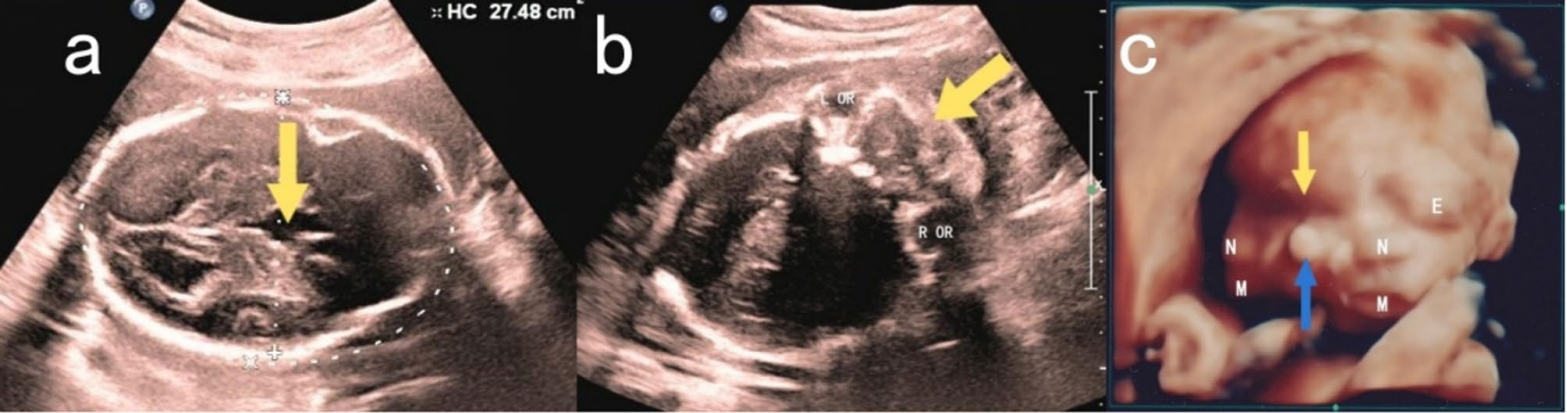
The coronal section through the thalamus demonstrates intact cranial bones, prominent and enlarged frontal lobes, and an indistinct corpus callosum. An anechoic area is observed at the midline of the brain as indicated by the arrow. (**b**)This image reveals abnormal widening of both orbits (OR: Orbit) with abnormal echogenicity between them as indicated by the arrow. (**c**) Three-dimensional surface imaging of the facial features confirms diprosopus, the fused orbits are indicated by the yellow arrow, and the fused auricles are indicated by the blue arrow (E: Eye; N: Nose; M: Mouth)

Subsequent ultrasound showed normal fetal growth except for head circumference (275 mm, > 98% Hadlock), which is abnormal (Table 1). Fetal heart rate was 124 bpm, with an anterior placenta (grade 0) and a deepest vertical pocket of amniotic fluid measuring 63 mm. Fetal cranial bones appeared intact, with frontal prominence and widened frontal lobes, and an indistinct distinction of the corpus callosum. An anechoic area was observed in the midline of the brain, measuring 13 mm x 9 mm (Fig. 1a), extending into the third ventricle, which appeared dilated by approximately 6 mm. The right lateral ventricle measured approximately 8.9 mm in width, and the left lateral ventricle (non-standard plane) measured approximately 9.7 mm in width. There were no obvious abnormalities in the cerebellum or posterior fossa. Both eye sockets were significantly widened, with disorganized echoes between them (Fig. 1b). Continuous sagittal scans of the facial region revealed the presence of two noses and two mouths. Three-dimensional surface imaging of the facial features revealed the presence of three eye sockets (the lateral eye sockets contained eyeballs, while the central region exhibited fusion without visible eyeballs), two noses, and two mouths (Fig. 1c). Fetal spine, heart, limbs, lungs, and abdominal organs appeared normal. Referring to the ultrasonic diagnostic features of diprosopus in fetuses described in previous literature, a comprehensive assessment led to the diagnosis of diprosopus. Additionally, abnormalities in intracranial structures, such as frontal lobe protrusion, unclear display of the corpus callosum, and anechoic areas in the midline extending to and dilating the third ventricle, also provided supplementary evidence for the diagnosis.

During the multidisciplinary prenatal consultation, they gained a comprehensive understanding of the fetus’s condition, possible prognosis, and subsequent management options. After careful consideration, they decided to terminate the pregnancy, taking into account the long-term health of the fetus and their family’s circumstances; however, they declined genetic testing.

The pregnant woman was admitted for induction of labor and signed the informed consent. Her vital signs and blood tests were normal. On the evening of Day 1, she received 75 mg of mifepristone. The following morning, on Day 2, she was given an additional 75 mg. Two hours later, 80 mg of ethacridine was injected into the amniotic cavity. After 12 h of labor, a female infant weighing 700 g and measuring 27 cm was successfully delivered. One minute after birth, the Apgar score was 2 (heart rate 60 bpm, weak respiration, very low muscle tone, reflexes not elicited, and cyanosis). The delivery process was smooth, with no perineal tears. Postpartum uterine contractions were adequate, and the placenta and membranes were delivered intact. Examination of the soft birth canal showed no injuries, with an estimated postpartum blood loss of approximately 150 ml of dark red blood and no clots. An oxytocin 20 U was administered intravenously to enhance contractions.

Unfortunately, due to the family’s refusal of further treatment, the newborn passed away 30 min later. The family hopes that sharing this case will provide insight into this rare condition and guidance for others in similar situations, which is why they signed the informed consent form.

Postpartum vital signs are normal, uterine contractions are good, and there were no adverse reactions. She was discharged on the third day postpartum.

**Table 1:** Fetal ultrasound measurement of development parameters

Development indicatorts	BPD	HC		AC	FL	HL	EFW	EGW
This case	70	275		213	45	41	875 ± 128 g	26 + 4 W
Hadlock	86%	>98%		19%	4%	13%	-	27 + 2 W

### Post-abortion examination

**Fig. 2:**
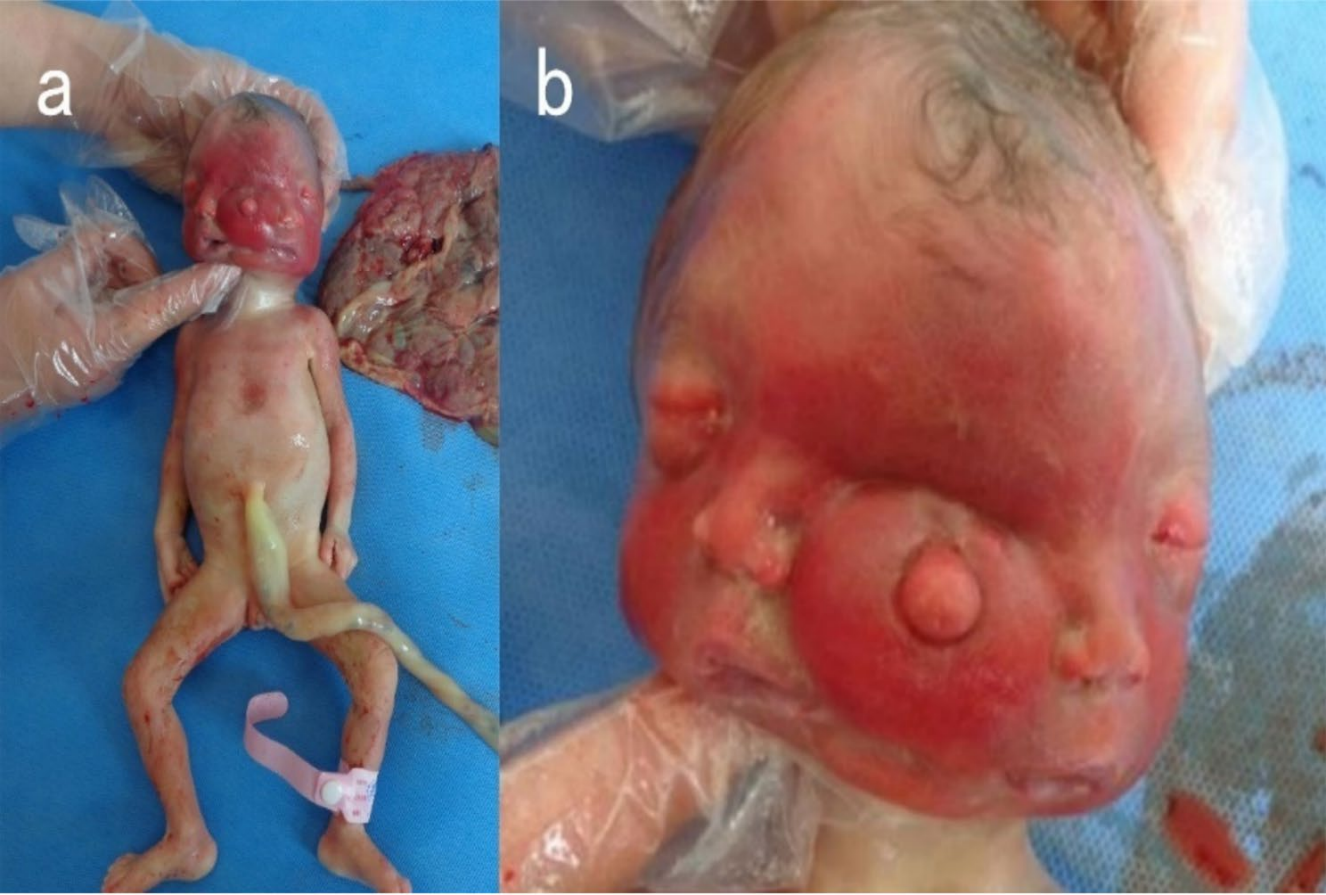
The specimen post-abortion shows (**a**) no abnormalities in the trunk and limbs, and (**b**) diprosopus in the facial features

**Fig. 3:**
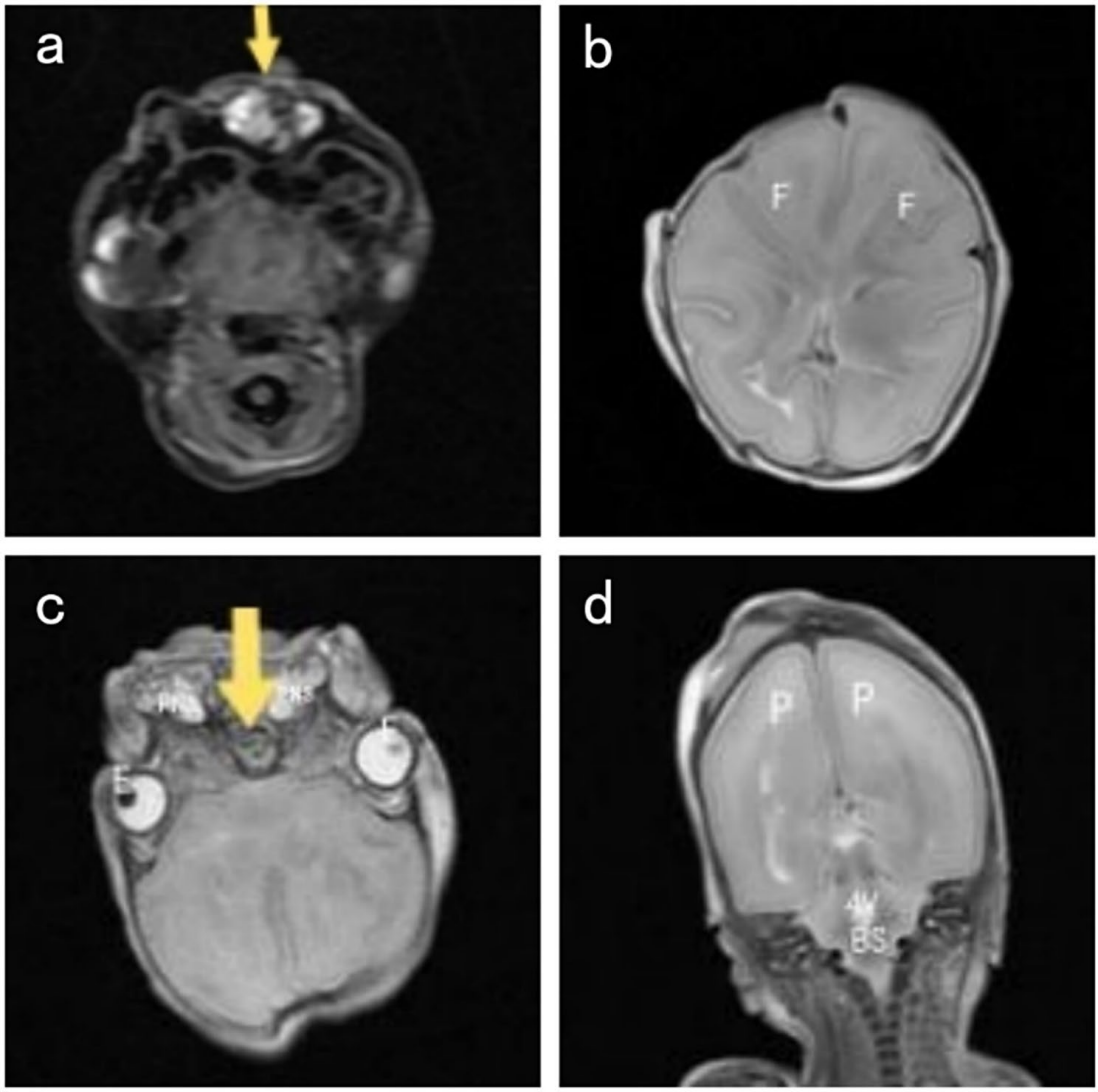
Postnatal MRI images of this case. (**a**) T1-weighted imaging reveals fusion of the bilateral maxillofacial bones as indicated by the arrow. (**b**) MRI T2-weighted imaging shows bilateral enlargement of the frontal lobes, widening of brain gyri, and an increased angle between the anterior edges of the lateral ventricles (F: Frontal Lobe). (**c**) T2-weighted imaging shows widening of the cranial bones in the frontal region, absence of eyeballs in the fused central eye region as indicated by the arrow, and two paranasal sinuses (E: Eye; PNS: Paranasal Sinuses). (**d**) T2-weighted imaging displays normal symmetric frontal and parietal lobes, brainstem, and fourth ventricle. (BS: Brain Stem; P: Parietal Lobe; 4 V: Fourth Ventricle)

**Fig. 4:**
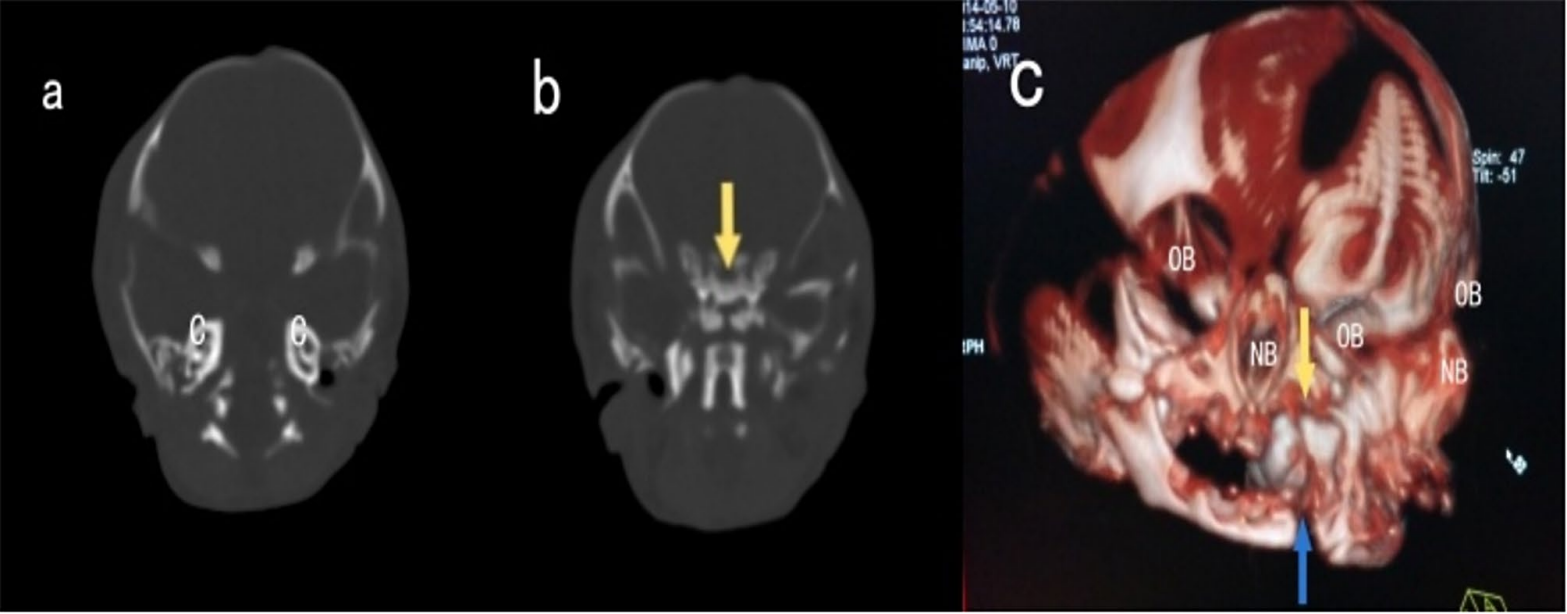
Postnatal CT images of this case (**a**) show two fully developed cochleae (C: Cochlea). (**b**) shows fusion of the two sphenoid bones, as indicated by the arrow in the middle (Sph: Sphenoid Bone). (**c**) CT three-dimensional imaging reveals the fusion of two nasal bones, two fused maxillae (indicated by the arrows) and two fused mandibles (indicated by the blue arrows), as well as three orbital bones (OB: Orbital Bones; NB: Nasal Bone)

Upon examination post-abortion, the fetus had a duplicated craniofacial structure with a similar appearance, including three eye sockets, two noses, two mouths, and two ears. Inside the lateral eye sockets, there were eyeballs, while the central region showed fusion without visible eyeballs. The upper and lower eyelids were tightly closed, with a protrusion at the site of facial fusion, possibly indicative of defective development of the earlobe, the appearance of the body below the neck was normal (Fig. 2). The MRI (Fig. 3) and CT (Fig. 4) scans performed on the day of induction show duplication and developmental abnormalities of the craniofacial structures in the frontal region. There were no abnormalities in the parietal and occipital lobes, cerebellum, or brainstem. Inside the protruding tissue at the central fusion point of the face, no structures resembling eyeballs were observed.

### Follow-up and outcomes

We conducted follow-up visits at one week, one month, and three months postpartum. The postpartum woman’s recovery was good, and psychological assessments indicated that she was adapting well and had stable emotions.

### Literature search

Through a search of published cases, we identified 16 cases of prenatal ultrasound-diagnosed diprosopus. We summarized the clinical and ultrasound findings from these cases (Tables 2 and 3). The maternal ages ranged from 13 to 33 years, and none of them had a relevant family history. One pregnant woman had a history of smoking, while another had a history of exposure to ampicillin, selexidine, and acrylic paint. Excluding two cases of unknown gender, the male-to-female ratio was 7:8. In addition to the typical bilateral features, 15 cases exhibited abnormalities in the central nervous system, including spinal cord defects and anencephaly. Seven cases had cardiovascular system abnormalities, such as Tetralogy of Fallot and transposition of the great arteries. Six cases showed respiratory system abnormalities, including lung hypoplasia. Two cases had gastrointestinal system abnormalities, including malrotation of the intestine and Meckel’s diverticulum. Two cases presented with genitourinary system abnormalities, including horseshoe kidney and small kidney. Additionally, there were other anomalies such as diaphragmatic hernia, single umbilical artery, undescended testes with micropenis, bilateral clubfeet, polydactylism, and more. Among the cases that underwent genetic testing, there was one abnormality (46XY, Y with a 983 kb deletion on chromosome 4q34.3, a 562 kb gain on chromosome Xp22.31p22.2, and a 32 kb gain on chromosome 13q12.11. Xp 22.31p22.2 and 13q12.11 were inherited from the mother), but there is no related information to prove its association with diprosopus [3].

The earliest detection of suspected diprosopus through 3D ultrasound occurred as early as the 12th week of gestation. Among the 16 cases, only one survived. In this surviving case, in addition to oral and lower jaw duplication malformation, no other abnormalities were observed.

**Table 2:** Clinal characteristics and pregnancy outcomes of all cases

Case No.	Maternal age(years)	Prece-ntage	Single or twin	Familial history of diprosopus	History of the exposure	Gestation & weight	Male or female	Pregnancy outcomes	Age at death
Case 1 [3]	29	G2P1	Single	-	-	36 W(2440 g)	Male	Death	0 days
Case 2 [4]	16	G1P0	Twin	-		33 W(1260 g)	Male	Death	stillbirth
Case 3 [5]	34	G4P-	Single	-	-	15 W(-g)	Female	Death	stillbirth
Case 4 [6]	26	G1P0	Twin	-	-	37 W(1800 g)	Male	Death	stillbirth
Case 5 [7]	31	G3P2	Single	-	+	40 W(3374 g)	Female	Live	-
Case 6 [8]	26	G1P0	Twin	-	-	37 W(1500 g)	Male	Death	0 days
Case 7 [9]	33	G1P1	Single	-	-	-W(450 g)	Male	Death	0 days
Case 8 [10]	27	G3P2	Single	-	-	20 W(-g)	Female	Death	stillbirth
Case 9 [11]	29	G2P1	Single	-	-	27 W(1090 g)	Female	Death	0 days
Case 10 [12]	28	G1P0	Single	-	-	12 W(-g)	-	Death	stillbirth
Case 11 [13]	22	G2P1	Single	-		12 W(-g)	Female	Death	stillbirth
Case 12 [14]	13	G1P0	Single	-	-	36 W(1700 g)	Female	Death	0 days
Case 13 [15]	27	G2P1	Single	-	+	20 W(-g)	Male	Death	stillbirth
Case 14 [16]	27	G2P1	Single	-	-	31 W(-g)	Female	Death	IFD
Case 15 [17]	30	G2P1	Single	-	-	13 W(-g)	Male	Death	stillbirth
Case 16 [18]	22	G2P1	Twin	-	-	28 W(1852 g)	Male	Death	1.5 days
This case	29	G1P0	Single	-	-	26 W(700 g)	Female	Death	stillbirth

The symbol “-” indicates the data previously not mentioned in the literature. W means week. History of the exposure including smoking, alcohol consumption, medical history, drug and supplementary intake, exposure to radiation, poison and chemicals and drug-exposure history. IFD: Intrauterine Fetal Demise

**Table 3:** Ultrasonic characteristics and post-abortion examination of 17 cases

Case No.	Number of					Abnormal of						AFV	NT	Other anomalies	DNA testing
	eyes	noses	mouths	ears	hypoplastic mandibles	CNS	CVS	RS	GI	GU	spine				
Case 1 [3]	4	2	2	2	2	**+**	**+**	**+**	**+**	**+**	-	-	-	CDH	**+**
Case 2 [4]	2^†^	2^†^	2^†^	2^†^	1	**+**	**+** ^**†**^	**+** ^**†**^	-	**+** ^**†**^	-	-	-	CDH^†^	-
Case 3 [5]	4^†^	2^†^	2^†^	2^†^	2	**+**	-	-	-	-	-	-	-	-	-
Case 4 [6]	4	2	2	3^†^	2	**+**	**+**	-	**+**	-	-	-	-	bilateral clubfeet	-
Case 5 [7]	2	1	2	2	2	-	-	-	-	-	-	-	-	-	-
Case 6 [8]	4	2	2	2	2	**+**	**+**	-	-	-	-	-	-	single umbilical artery; undescended testes and micropenis; bilateral clubfeet	-
Case 7 [9]	3	2	2	2	2	**+**	-	**+** ^**†**^	-	-	-	-	-	polydactylism	-
Case 8 [10]	4	2	2	3	2	**+**	-	-		-	-	**+**	-	CDH	-
Case 9 [11]	2	2	2	2	2	**+**	-	**+** ^**†**^	-	-	-	**+**		two splenules	-
Case 10 [12]	3	2^†^	2^†^	2^†^	-	**+**	-	-	-	-	-	-	-	-	-
Case 11 [13]	4	2	2	4	2	**+**	**+** ^**†**^	-	-	-	-	-	-	-	-
Case 12 [14]	4	2	2	2	2	**+**	**+**	-	-	-	**+**	**+**	-	CDH	-
Case 13 [15]	4	2	2	2	2	**+**	-	-	-	-	**+**			-	-
Case 14 [16]	4	2	2	2	2	**+**	-	-	-	-	-	**+**	-	-	-
Case 15 [17]	4^†^	2^†^	2^†^	2^†^	-	**+**	-	-	-	-	-	-	-	gastroschisis	-
Case 16 [18]	4	2	2	-	2	**+**	**+**	-	-	-	-	**+**	-	-	-
This case	3	2	2	3	2	**+**	-	-	-	-	-	-	-	-	-

The symbol “-” indicates data or negative results that were not mentioned previously in the literature. The symbol “^†^” indicates postnatal examination revealed abnormalities. CNS: Central Nervous System, CVS: Cardiovascular System, RS: Respiratory System, GI: Gastrointestinal System, GU: Genitourinary System, AFV: Amniotic Fluid Volume, NT: Nuchal Translucency; CDH: Congenital Diaphragmatic Hernia

## Discussion

In this case, we observed a severe presentation of diprosopus in the fetus, characterized by two complete faces, four eyes, one of which was fused and lacked a developed eyeball. Additionally, there were absent ears at the site of facial fusion, forming a prominent protuberance. Notably, the anatomy below the neck appeared normal, with no evident abnormalities.

Through our review of published cases, we noted that diprosopus typically presents with facial duplication, characterized by an increased number of eyes, noses, mouths, lower jaws, and ears [8–14]. Most cases are associated with central nervous system abnormalities, and cardiovascular structural defects are also common [6, 8, 14, 18]. The mortality rate for fetuses with diprosopus is high. However, some studies indicate that isolated lower jaw and oral duplications may have a more favorable prognosis [19].

In our case, the initial ultrasound clues included significant widening of the eye sockets and disorganized echoes between them, making it challenging to distinguish from a central facial mass lesion. Continuous sagittal scans of the facial region revealed two nasal contours, confirming the presence of bilateral noses and lips. Subsequent three-dimensional imaging clearly demonstrated the bilateral craniofacial appearance, highlighting that a simple two-dimensional examination may be inadequate and prone to missed diagnoses. Additionally, a head circumference measurement of 275 mm, exceeding the 98th percentile for the gestational age, along with significant frontal widening, raised suspicion and warranted further MRI examination.

The incidence of diprosopus is estimated to be 1.4 per million births [2]. The etiology remains unclear, although several hypotheses have been proposed. One theory posits that diprosopus arises from conjoined twinning, involving the fusion of two primitive nodes before notochord formation, fusion of two embryonic discs leading to developmental anomalies, and splitting of the neural crest into two notochords [3]. Additionally, abnormalities in Sonic Hedgehog (Shh) genes and proteins are widely accepted as contributing factors. These abnormalities can disrupt signaling and patterning essential for craniofacial development, organizing embryonic cells into regions that later develop into specific organs. For instance, a deficiency in Shh protein may lead to holoprosencephaly, while ectopic movement of the optic discs can result in various eye abnormalities. Increased activity of Shh can cause organ duplications, such as diprosopus. Other theories suggest that fusion of parallel notochords or abnormalities in DIX homologous box genes might lead to the splitting of a single notochord. However, no specific genetic abnormalities linked to diprosopus have been documented to date [3].

Prenatal 3D ultrasound imaging effectively demonstrated diprosopus and suggested intracranial structural abnormalities. Post-abortion clinical presentation, along with MRI and CT imaging, indicated partial duplication and developmental abnormalities of cranial structures, consistent with the prenatal ultrasound findings. Unfortunately, the postpartum woman declined chromosomal testing, preventing confirmation of any chromosomal abnormalities associated with the diprosopus.

## Conclusion

Abnormalities in the frontal brain structure, increased abnormal eye distance, and enlarged head circumference are potential clues for prenatal ultrasound screening of diprosopus. Furthermore, conducting a continuous multi-sectional scan of the facial region can reveal more clues, while three-dimensional facial imaging provides an intuitive display to allows for a multi-angle observation and clear visualization of the bilateral facial appearance, facilitating a definitive diagnosis of diprosopus. Early diagnosis of diprosopus and multidisciplinary prenatal counseling are of great significance for individualized management of pregnant women, psychological support, and, when necessary, the termination of pregnancy.

## Electronic supplementary material

Below is the link to the electronic supplementary material.

Supplementary Material 1

## Data Availability

No datasets were generated or analysed during the current study.

## Abbreviations

2D ultrasound: Two-Dimensional Ultrasound
3D ultrasound: Three-Dimensional Ultrasound
MCMA: Monoamniotic-monochorionic
IFD: Intrauterine Fetal Demise
CNS: Central Nervous System
CVS: Cardiovascular System
RS: Respiratory System
GI: Gastrointestinal System
GU: Genitourinary System
AFV: Amniotic Fluid Volume
NT: Nuchal Translucency
CDH: Congenital Diaphragmatic Hernia
