# Supplementary material for: Three-dimensional sonographic findings of diprosopus: a case report and literature review
Source: BMC Pregnancy Childbirth. 2025 Jan 20;25:43. doi: 10.1186/s12884-025-07168-0 (PMC11744991; doi:10.1186/s12884-025-07168-0)
Supplement: Supplementary file 1 — Supplementary Material 1 [file 12884_2025_7168_MOESM1_ESM.docx]

Timeline Summary

| Time Point | Event |
| --- | --- |
| 6 weeks | Normal prenatal check-up |
| 12 weeks | Normal prenatal check-up |
| 17 weeks | Normal prenatal check-up |
| 24 weeks | Facial abnormalities detected during check-up |
| 26 weeks | Abnormalities noted during this check-up |

*Note: The article provides a detailed description of the facial abnormalities at 26 weeks.*
